# Golgi Cell-Mediated Activation of Postsynaptic GABA_B_ Receptors Induces Disinhibition of the Golgi Cell-Granule Cell Synapse in Rat Cerebellum

**DOI:** 10.1371/journal.pone.0043417

**Published:** 2012-08-22

**Authors:** Federico Brandalise, Urs Gerber, Paola Rossi

**Affiliations:** 1 Dipartimento di Biologia e Biotecnologie “L. Spallanzani”, University of Pavia, Pavia, Italy; 2 Brain Research Institute, University of Zurich, Zurich, Switzerland; Baylor College of Medicine, United States of America

## Abstract

In the cerebellar glomerulus, GABAergic synapses formed by Golgi cells regulate excitatory transmission from mossy fibers to granule cells through feed-forward and feedback mechanisms. In acute cerebellar slices, we found that stimulating Golgi cell axons with a train of 10 impulses at 100 Hz transiently inhibited both the phasic and the tonic components of inhibitory responses recorded in granule cells. This effect was blocked by the GABA_B_ receptor blocker CGP35348, and could be mimicked by bath-application of baclofen (30 µM). This depression of IPSCs was prevented when granule cells were dialyzed with GDPβS. Furthermore, when synaptic transmission was blocked, GABA_A_ currents induced in granule cells by localized muscimol application were inhibited by the GABA_B_ receptor agonist baclofen. These findings indicate that postsynaptic GABA_B_ receptors are primarily responsible for the depression of IPSCs. This inhibition of inhibitory events results in an unexpected excitatory action by Golgi cells on granule cell targets. The reduction of Golgi cell-mediated inhibition in the cerebellar glomerulus may represent a regulatory mechanism to shift the balance between excitation and inhibition in the glomerulus during cerebellar information processing.

## Introduction

The cerebellum is a brain structure important for the precise execution of motor sequences. It performs critical functions required for error or novelty detection by processing differences between predictions elaborated by the cortex and incoming stimuli conveyed by the senses.

Different parts of cerebellum fulfill distinct physiological functions. The vestibulo-cerebellum, constituted by the flocculo-nodular lobe and adjacent vermis, regulates equilibrium and vestibulo-ocular reflexes. The spino-cerebellum, including the vermis and the intermediate part of hemispheres, is involved in movement execution including feedback adjustments. The cerebro-cerebellum, represented by the lateral part of the cerebellar hemispheres, plays an important role in preparation, initiation and timing of motor acts via the dentate nuclei.

Cerebellar networks can be subdivided into three layers: an input (granular) layer, an intermediate processing (molecular) layer and an output (Purkinje) layer connected to the deep cerebellar nuclei. The granular layer and the molecular layer form the cortical part of the cerebellum. The deep cerebellar nuclei complex, which is part of the precerebellar nuclei, represents the only output pathway of the cerebellar cortex. The granular layer is composed of three main classes of neurons: granule cells, Golgi cells, and Lugaro cells. In the vestibular cerebellum, a fourth neuron type is represented by the unipolar brush cell (UBC). The mossy fibers make excitatory glutamatergic synapses with all these cell types. The Golgi cells make inhibitory connection to granule cells and UBCs and the UBCs inhibit Golgi cells. The granule cells send excitatory inputs to the Purkinje cells and to molecular layer interneurons. In turn, the Golgi cells provide the only inhibitory input to the granular layer, generating a complex combination of feed-forward, feed-back and lateral inhibition responses.

Golgi cells are GABAergic interneurons that modulate transmission through the cerebellar glomerulus, thereby regulating the input-output relationship and the gain at the synapses between mossy fibers and granule cells [1], [2]. Thus, Golgi cells do not simply inhibit granule cells. For example, an *in vivo* investigation in the ventral paraflocculus (VPFL) of the alert squirrel monkey has shown that Golgi cells operate as state-specific temporal filters at the mossy fiber-granule cell input during a variety of vestibular and oculomotor behaviors [3]. Furthermore, a paradoxical excitatory action has been reported at the Golgi cell – granule cell synapse mediated by presynaptic metabotropic glutamate receptors [4]. A number of *in vitro* studies have also characterized novel properties of Golgi cell function that challenge the classical view of their roles in regulating transmission to the cerebellar cortex. Golgi cell discharge not only evokes synaptic IPSCs but also generates pronounced tonic inhibition [5], [6], [7]. This tonic response reflects the activation by GABA spillover of high affinity extrasynaptic receptors containing the α6 subunit, and the accumulation of ambient GABA at submicromolar concentrations in the glomerulus [8], [9], [10]. In addition to the GABA_A_ receptors expressed by granule cells, the glomerulus also contains GABA_B_ receptors localized in the somatodendritic compartment of granule cells and on the terminals of Golgi cells [11]. The postsynaptic GABA_B_ receptors on cerebellar granule cells have been shown to mediate inhibition of a rectifier current [12]. The GABA_B_ receptors on Golgi cell terminals, which exhibit high affinity, are tonically activated by ambient GABA [13] resulting in a decrease in release probability at the onset of Golgi cell discharge and thus in a modulation of inhibitory signaling.

In the present study, we investigated whether phasic increases in GABA release are also capable of modulating inhibitory synaptic transmission. Following the stimulation of Golgi cell axons with a brief train of high frequency pulses (100 Hz), comparable to the frequency of *in vivo* activity recorded in Golgi cells [14], [15], [16], we observed an inhibition of both phasic and tonic GABA_A_ receptor-mediated responses. Interestingly, we find that this phenomenon depends primarily on the activation of postsynaptic GABA_B_ receptors of granule cells.

**Figure 1:**
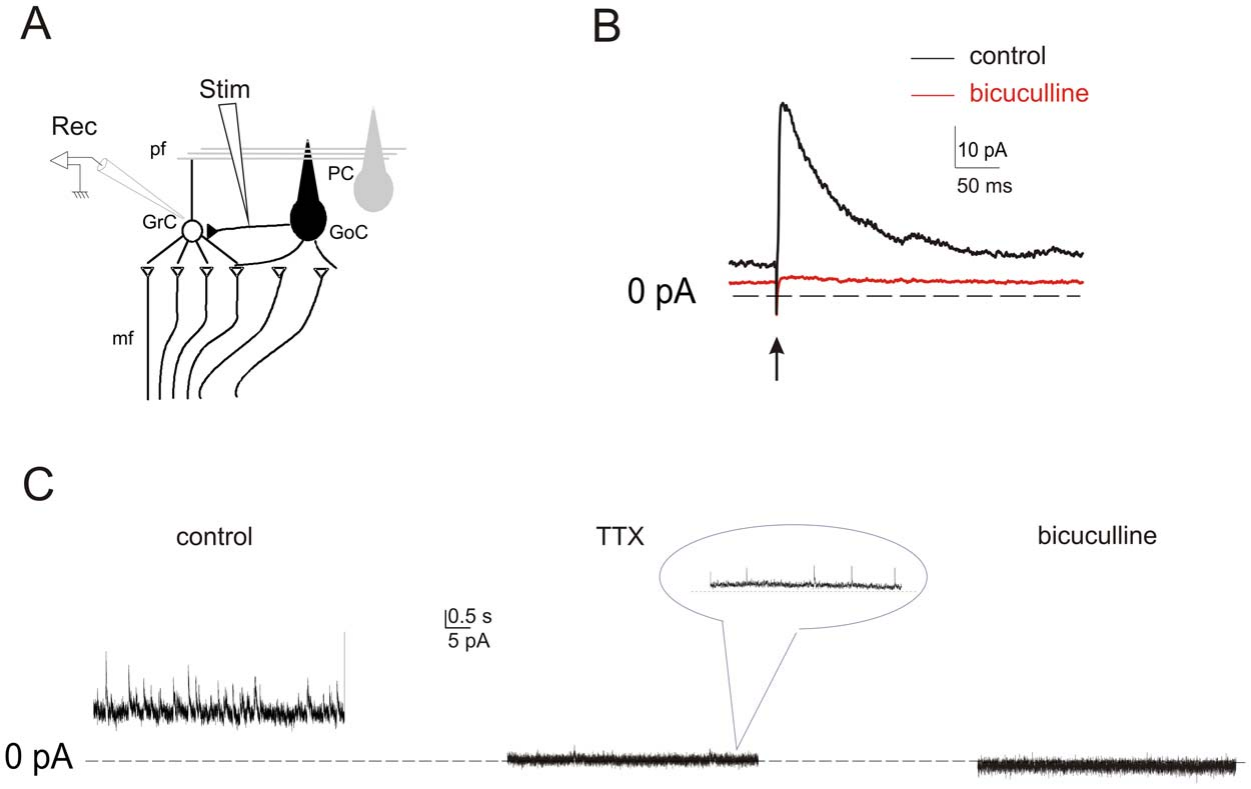
Schematic of the experimental conditions. ***A,*** In the cerebellar glomerulus, Golgi cells (black) release GABA onto mossy fiber terminals as well as their targeted granule cells. In these experiments, Golgi cell axons are electrically stimulated and evoked IPSCs are recorded in a patch-clamped granule cell. Ionotropic and metabotropic glutamate receptors are blocked pharmacologically with D-APV, 7-Cl-kainate, CNQX, and AIDA to prevent excitatory transmission. ***B,*** Averaged eIPSCs recorded in cerebellar granule cells by Golgi cell axon stimulation (arrow) before (control) and after 10 µM bicuculline (*bicuculline*) local perfusion. The arrow indicates the time of stimulation. ***C,*** Tonic and phasic current were abolished by 1 µM TTX (*TTX)* leaving rare miniature spontaneous IPSCs (*minis*, magnified in the inset; vertical scale bar corresponds to 5 pA; horizontal scale bar corresponds to 1 s). Note also that background noise decreased during TTX perfusion. Subsequent application of 10 µM bicuculline.

## Methods

### Preparation of Brain Slices and Solutions for Recordings

Patch-clamp recordings in acute cerebellar slices were obtained as previously described [12], [17], [18], [19]. All experimental procedures were approved by the Ethics Committee of the University of Pavia. Briefly, 17–23 day-old Wistar rats were anesthetized with halothane inhalation (Aldrich, Milwaukee, WI), beheaded, and the cerebellar vermis was dissected out and placed into ice-cold Krebs solution. Vibratome sagittal sections from cerebellar vermis (220 µm thickness) were allowed to recover for at least 30 min at room temperature in Krebs solution continually bubbled with oxygen/carbon dioxide (95%/5%) before being transferred to a 1.5 ml recording chamber mounted on the stage of an upright microscope (OLYMPUS BX51WI, Japan). Slices were superfused with Krebs solution (flow rate 2 ml/min) and maintained at 30°C with a Peltier-feedback device (TC-324B, Warner Instr. Corp. Hamden, CT, USA). Krebs solution for slice cutting and recovery contained (in mM): NaCl 120, KCl 2, MgSO_4_ 1.2, NaHCO_3_ 26, KH_2_PO_4_ 1.2, CaCl_2_ 2, glucose 11, and was equilibrated with 95% O_2_ and 5% CO_2_ (pH 7.4). The n value referring to the number of the cells and, on average we recorded two cells per animal.

**Figure 2:**
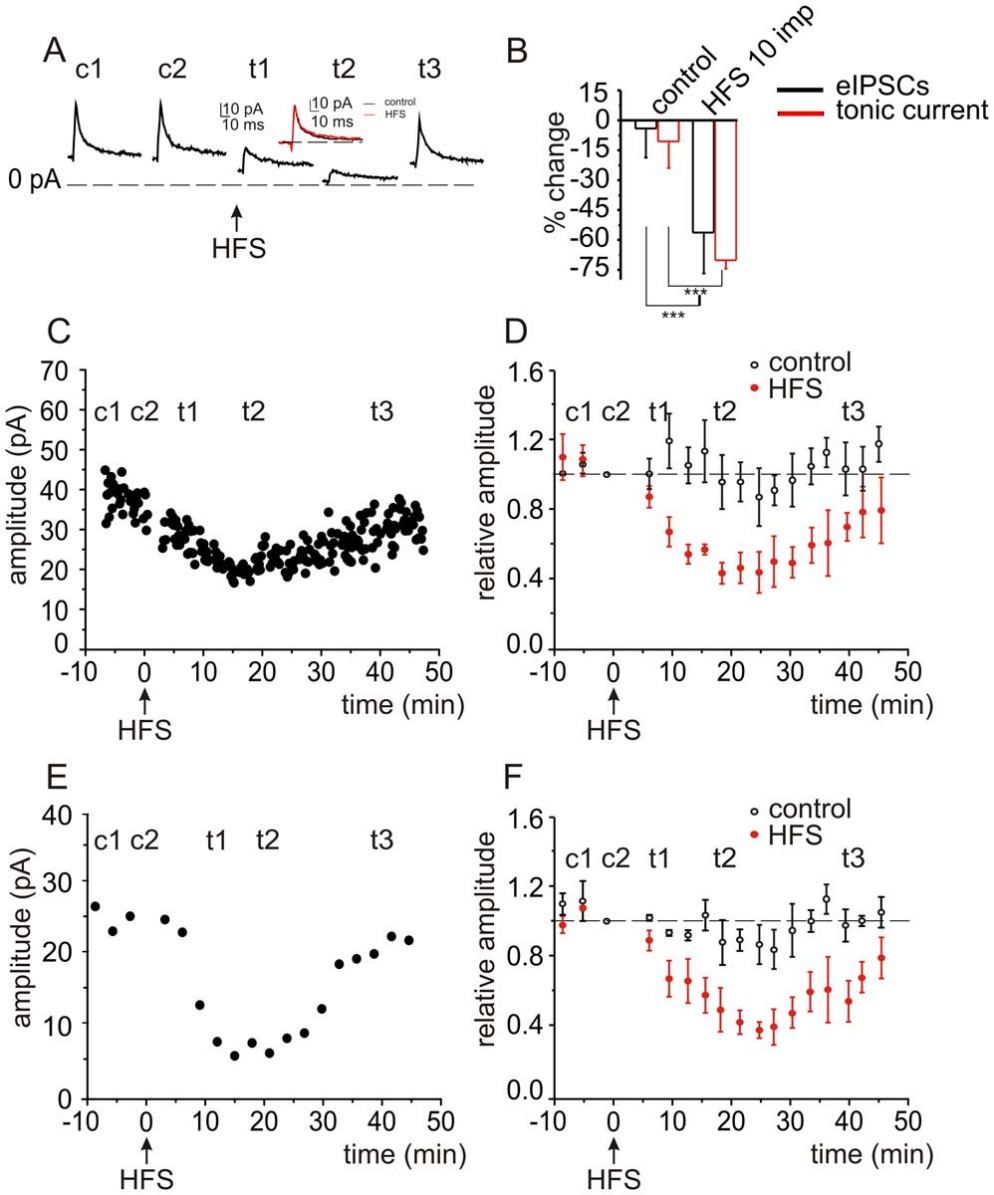
GABAergic signaling at the Golgi cell – granule cell synapse is transiently inhibited following high frequency stimulation. *A,* GABAergic IPSCs were evoked in granule cells by stimulating Golgi cell axons at 0.1 Hz. High frequency stimulation (HFS, 10 pulses at 100 Hz) reduced both eIPSCs and I_tonic_ current. Depicted responses are averages of 5 to 10 eIPSCs, before (c1, c2) and after HFS, at 5 min (t1), 20 min (t2), and 40 min (t3). Inset shows superimposed traces of c1 and t1 scaled to the same amplitude indicating that the kinetics of the response were not changed. ***B,*** Histograms show averaged data from 12 cells for eIPSCs (black) and 10 cells for I_tonic_ (red). ***C,*** Time course of the changes in eIPSC amplitude following HFS for a representative cell, and ***D,*** averaged responses from 12 cells. ***E,*** Time course of the changes in I_tonic_ for a single cell, and ***F*** **,** averaged values for 10 cells.

### Whole-cell Patch-clamp Recordings

We recorded spontaneous inhibitory postsynaptic currents (*sIPSCs*), evoked inhibitory postsynaptic currents (*eIPSCs*) and I_tonic_ from cerebellar granule cells using the blind whole-cell patch-clamp recording technique [20]. Recordings were obtained from lobule VI of the cerebellar vermis.

**Figure 3:**
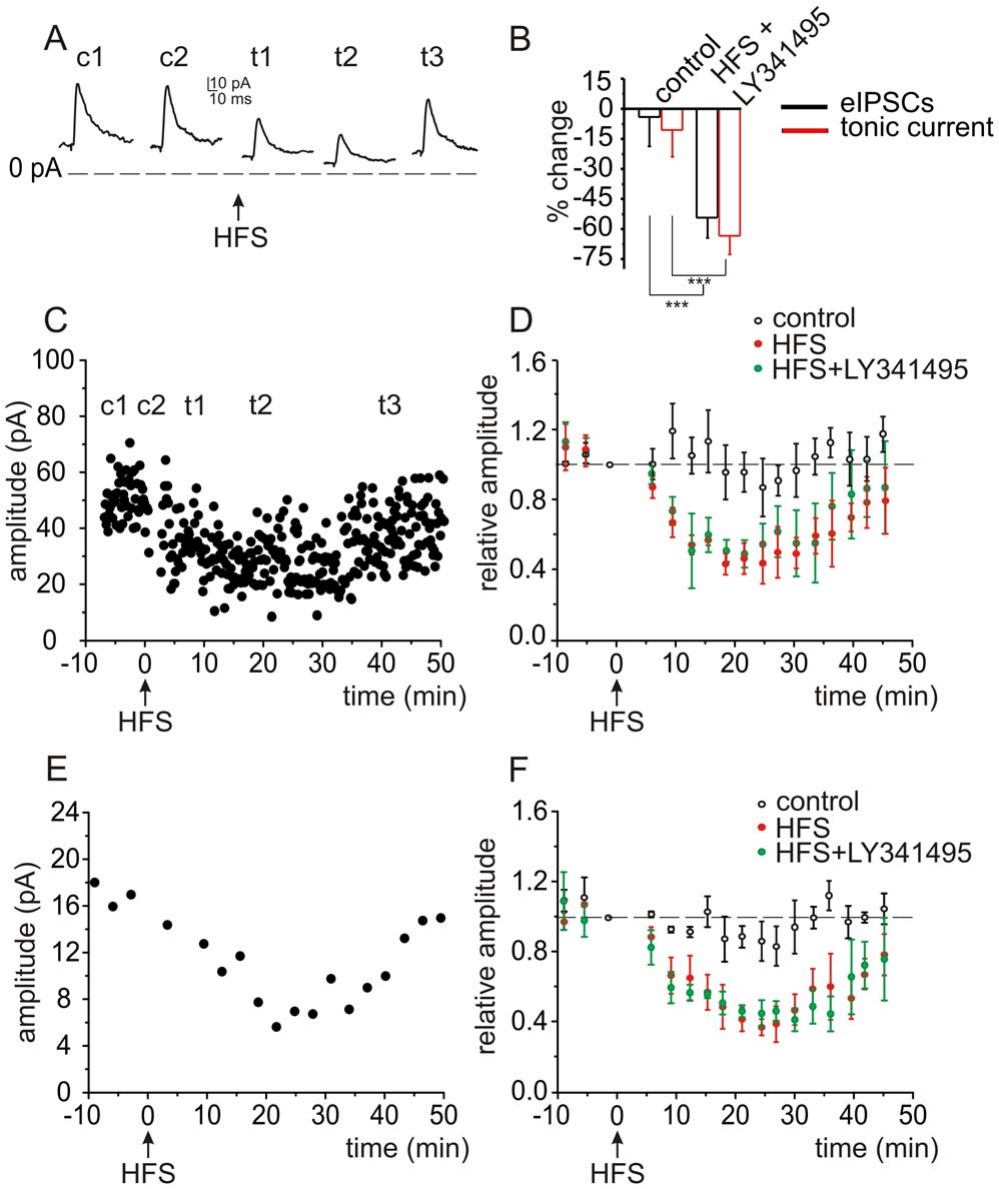
GABAergic signaling at the Golgi cell – granule cell synapse is still transiently inhibited following high frequency stimulation when mGluRs are blocked. *A,* GABAergic IPSCs were evoked in granule cells by stimulating Golgi cell axons at 0.1 Hz. After including LY341495 (100 µM) in the cocktail of antagonists (see Methods), high frequency stimulation (HFS, 10 pulses at 100 Hz) still reduced both eIPSCs and I_tonic_ current. Depicted responses are averages of 5 to 10 eIPSCs, before (c1, c2) and after HFS, at 5 min (t1), 20 min (t2), and 40 min (t3). ***B*** **,** Histograms show averaged data from 4 cells for eIPSCs (black) and 4 cells for I_tonic_ (red). ***C,*** Time course of the changes in eIPSC amplitude following HFS for a representative cell, and ***D,*** Histograms showing averaged data from 4 cells for eIPSCs compared to data from the same experiment without LY341495 (Fig. 2). ***E,*** Time course of the changes in I_tonic_ for a single cell, and ***F,*** averaged values for 4 cells in comparison to the same experiment without LY341495.

A 200B amplifier was interfaced to pClamp command/record software through a Digidata 1440A analog/digital converter (Axon Instruments; low-pass filter = 10 kHz, sampling rate = 100 kHz**)**. Recording electrodes (5–8 MΩ) were filled with (in mM) Cs_2_SO_4_ 81, NaCl 10, MgSO_4_ 2, CaCl_2_ 0.02, BAPTA 0.1, glucose 15, ATP-Mg 3, GTP 0.1, HEPES 15 (pH was adjusted to 7.2 with CsOH; resting free [Ca^2+^] 100 nM).This intracellular solution provides a physiological chloride gradient (chloride reversal potential: E_Cl_ = −66 mV; 5,9). Cesium was chosen to exclude potential effects of postsynaptic GABA_B_ receptors on K^+^-currents [12]. In some experiments we added guanosine-5′-O-(2-thiodiphosphate) (GDPβS) at a concentration of 1 mM to the standard pipette solution. Granule cells were voltage-clamped at holding potential of −10 mV where the inward Cl^-^ current is recorded as an outward positive current.

**Figure 4:**
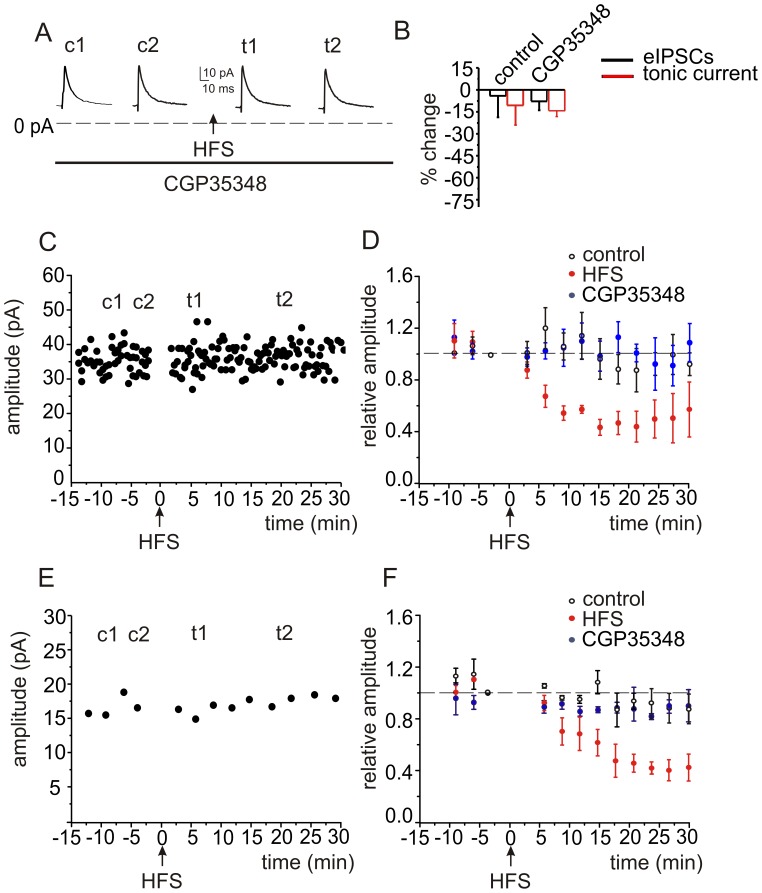
Golgi cell-mediated inhibition of GABA_A_ responses in granule cells is prevented in the presence of a GABA_B_ receptor antagonist. *A,* Preincubation and perfusion of the GABA_B_ receptor antagonist CGP35348 (50 µM) blocked the effects of HFS on phasic and I_tonic_ GABA_A_ receptor mediated responses. ***B,*** Histograms show averaged data for eIPSCs (black) and I_tonic_ (red). The differences in the averaged data are not significant. ***C,*** An example of the time course of responses from one experiment performed with CGP35348, and ***D,*** averaged time course of eIPSCs (n = 12). For comparison the averaged time course from control recordings without HFS (control), and the averaged time course with HFS (Fig. 2D) are also shown. ***E,*** Time course of the changes in I_tonic_ for a single cell, and, ***F,*** averaged values for all cells (n = 12).

### Perforated Patch-clamp Recordings

We also recorded inhibitory currents from cerebellar granule cells using the perforated patch-clamp recording technique [21]. The recordings were obtained with 5–8 MΩ electrodes filled with a solution containing nystatin (100 µg ml^−1^) and (mM) K_2_SO_4_ 84, NaCl 10, glucose 30, HEPES buffer 5 (pH was adjusted to 7.2 with KOH). The series resistance averaged 23.6±4 MΩ.

**Figure 5:**
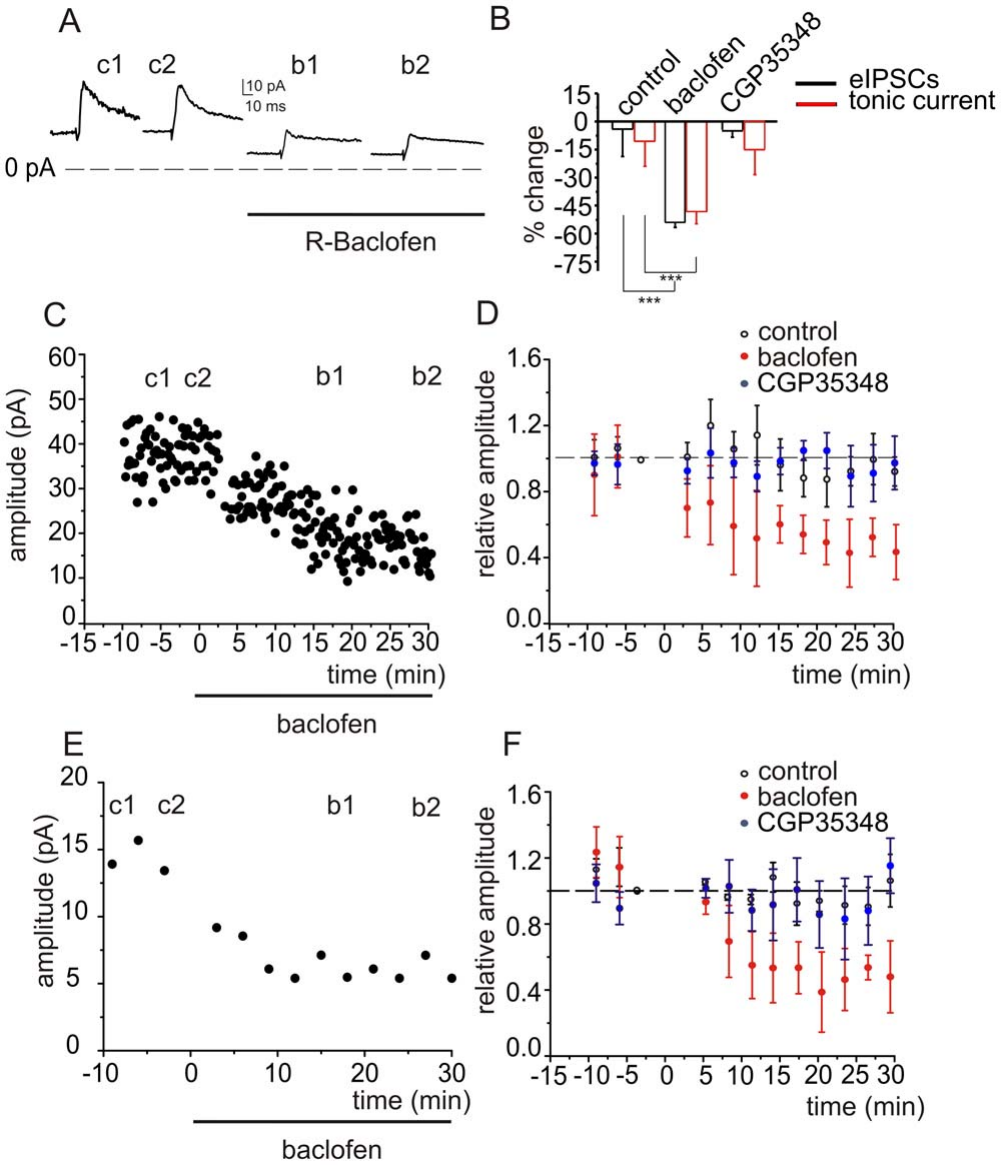
Pharmacological activation of GABA_B_ receptors depresses GABA_A_ receptor-mediated responses in granule cells. ***A,*** The traces of averaged eIPSCs evoked at 0.1 Hz show that local application of baclofen (30 µM, 30 min) reduces both eIPSCs and outward I_tonic_. ***B,*** Histograms show averaged data from 8 cells for eIPSCs (black) and I_tonic_ (red). ***C,*** Representative experiment showing the time course of the effect of baclofen. ***D,*** Averaged data for 8 cells. ***E,*** Time course of the effect of baclofen on I_tonic_ for a single cell, and, ***F,*** averaged values for all 8 cells.

### Golgi Cell Axon Stimulation

Golgi cell axons were stimulated with a glass pipette through an isolation unit. The minimal stimulation intensity was determined to be ∼ 8 V by comparing evoked IPSPs with spontaneous IPSPs. Experiments were then performed with suprathreshold strength by increasing the stimulation intensity by 20%.

### Drug Perfusion

R-Baclofen and CGP35348 were applied to the bath in some experiments, and by local perfusion through a multi-barreled glass pipette positioned close to (about 50 µm) the recorded cells in other experiments. The concentrations indicated for local perfusion are concentrations in the pipette. CNQX (10 µM), D-APV (100 µM), 7Cl-Kyn (50 µM), AIDA (50 µM), bicuculline (10 µM) and LY341495 (100 µM) were obtained from Tocris-Cookson, Avonmouth, UK and tetrodotoxin (TTX) from LATOXAN, Valence, France. Local perfusion with Krebs solution and glutamate antagonists was commenced before seal formation and was maintained until switching to the test solutions.

**Figure 6:**
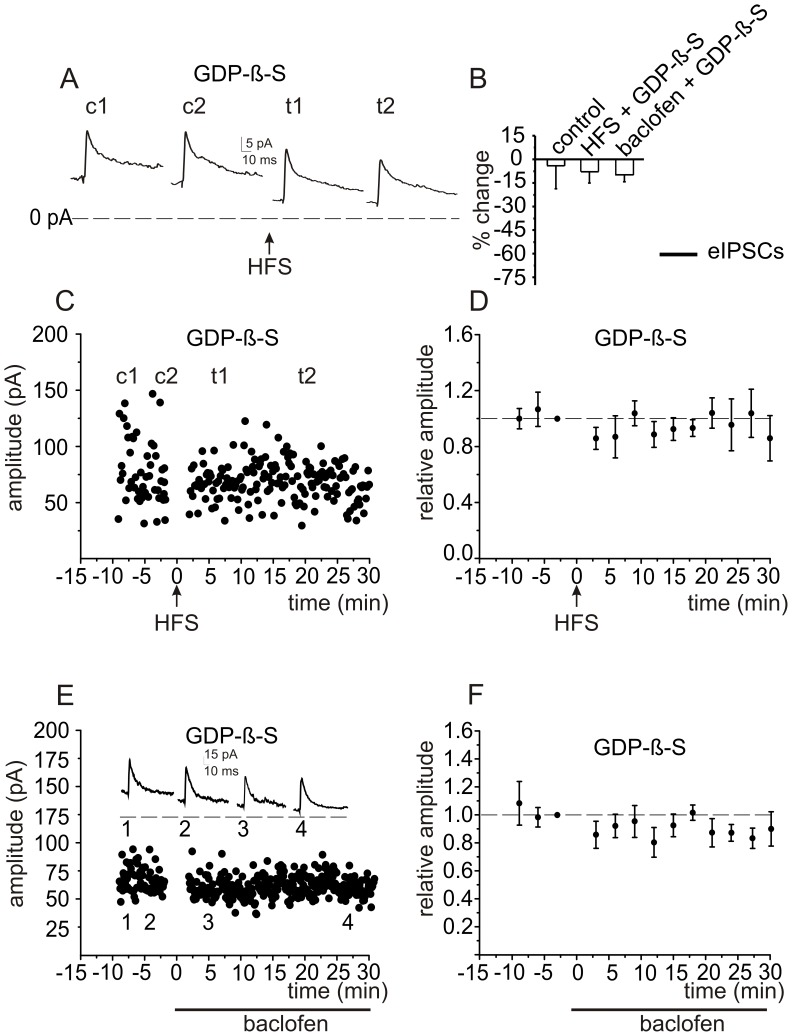
Inhibiting G-protein function in the postsynaptic granule cell blocks the effect of HFS or baclofen on eIPSCs. *A,* Inhibiting G-protein function in the postsynaptic granule cell blocks the effect of HFS on eIPSCs**.** eIPSCs were recorded in GCs following intracellular dialysis of GDPβS (1 mM) to block G-protein function. Averaged eIPSC traces are shown at 1 min (c1) and 5 min (c2) after establishing the whole cell configuration and at 5 min (t1) and 20 min (t2) after HFS. ***B,*** Histograms show averaged data for the effects of HFS (n = 5) and baclofen (n = 4) on eIPSCs. ***C,*** Time course of responses from a single cell, and ***D,*** pooled data in the presence of intracellular GDPβS before and after HFS. ***E,*** Inhibiting G-protein function in the postsynaptic granule cell blocks the effect of baclofen on eIPSCs in a single cell, and ***F,*** in pooled data for 4 cells.

### Acquisition and Analysis

IPSCs were digitally filtered at 1.5 kHz and analyzed off-line with pCLAMP8 (Axon Instruments). Peak amplitude and decay time constants were analyzed. Series resistance was monitored by measuring passive current transients induced by 10 mV hyperpolarizing voltage steps from a holding potential of −60 mV. Cerebellar granule cells have a compact structure and behave like a single electrotonic compartment [17], [18], [22], [23]. Accordingly, the transients were reliably fitted with a mono-exponential function yielding a membrane capacitance of C_m_ = 4.4±0.3 pF, membrane resistance R_m_ = 2.8±0.4 GΩ and series resistance R_s_ = 13.5±1.3 MΩ (n = 29). During whole-cell recordings capacitative transients were measured to monitor potential changes in series resistance. The −3 dB cell + electrode cut-off frequency was f_VC_ = (2πR_s_C_m_)^−1^ = 3.1±0.2 kHz (n = 29). Accepted deviations from these parameters in current transients recorded over the time-windows used for statistical analysis were less than 10%. The arrows indicate the time of stimulation, defined as time 0 on the time scale. Experimental traces are shown without baseline adjustments: dashed lines indicate the 0 pA level. Where indicated, relative amplitude was calculated by normalizing data just before HFS application.

**Figure 7:**
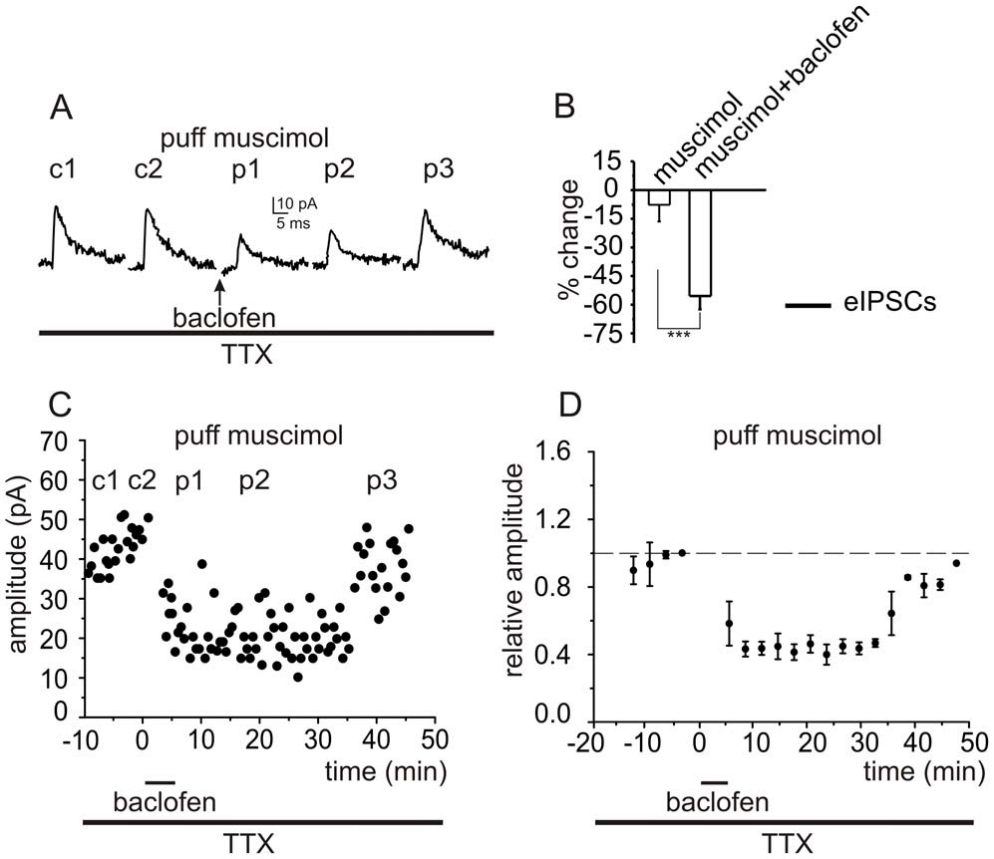
Activation of postsynaptic GABA_B_ receptors is sufficient to inhibit GABA_A_ receptor-mediated responses in granule cells. *A,* To examine the effects of GABA_B_ receptor activation in a granule cell in the absence of Golgi cell stimulation, synaptic transmission was blocked by superfusing slices with TTX (1 µM) and GABA_A_ receptor-mediated responses were elicited by puff applications of muscimol. Averaged responses to muscimol are shown at 1 min (c1) and 5 min (c2) after establishing the whole cell configuration and at 10 min (p1), 20 min (p2), and 40- min (p3) after bath-application of baclofen. ***B*** **,** Histograms show averaged data for the effects of baclofen on muscimol-induced responses (n = 4). ***C,*** Time course of responses from a single cell, and ***D,*** pooled data for the effect of baclofen on muscimol-induced GABA_A_ receptor-mediated currents.

Data are reported as means±SEM and statistical comparisons were done using Student’s paired *t*-test. The mean % change reported for different experimental conditions was calculated by averaging values from single cells. Traces were averaged digitally for 5 to 10 IPSCs. Averaged time courses are the means from different cells as indicated in the legends.

## Results

### High Frequency Stimulation of Golgi Cell Axons Induces Transient Inhibition of Phasic and Tonic GABAergic Responses in Granule Cells

To determine whether brief discharge of Golgi cells modulates inhibitory transmission at the Golgi cell–granule cell synapse, we stimulated Golgi cell axons with an electrode positioned in the granular cell layer, close (<100 µm) to a voltage-clamped granule cell. In all experiments, inhibitory responses were pharmacologically isolated by superfusing the slice with antagonists for ionotropic (CNQX, 10 µM; D-APV, 100 µM; 7Cl-Kyn, 50 µM) and metabotropic (AIDA, 50 µM) glutamate receptors (Fig. 1). Golgi cell axons were stimulated at a basal frequency of 0.1 Hz using a suprathreshold intensity, which at holding potential of −10 mV evoked eIPSCs occurring with a delay of 2.2±0.16 ms, consistent with a monosynaptic response (n = 24; Fig. 2*A*). All evoked activity was abolished with application of 1 µM tetrodotoxin (TTX) or 10 µM bicuculline (Fig. 1*C*) indicating that the responses were synaptic and mediated by GABA_A_ receptors. These results confirm the absence of slow GABA_B_ receptor-mediated responses in phasic granule cell inhibitory currents [12], [13]. Beside affecting synaptic currents, bicuculline and TTX caused an inward current shift revealing the presence of a tonic GABA_A_ receptor-mediated leak conductance accompanied by a decrease in background noise due to reduced stochastic channel openings [9], [10], [24], [25], [26]. Local perfusion of bicuculline caused an inward current shift of 13.4 pA corresponding to a conductance change of 239.2±38.6 pS (n = 8, P<0.001 paired *t-*test, Fig. 1*B*). TTX caused an inward current shift of 10.9 pA corresponding to a conductance change of 194.6±32.2 pS (n = 20, P<0.00019 unpaired *t-*test, Fig. 1*C*). After TTX application, subsequent application of bicuculline (Fig. 1*C*) caused a non-statistically significant current shift (n = 5, Fig. 1*C*). Note the rare TTX-insensitive spontaneous events (Fig. 1*C*, inset in center panel) that were identified as inhibitory miniature synaptic currents (*minis*). These effects match previous reports of bicuculline-sensitive tonic inhibition in granule cells, which includes a conspicuous component sustained by spontaneous Golgi cell autorhythmic activity.

After a 10 min control period we delivered a train of high frequency stimulation (HFS, Fig. 2) consisting of 10 impulses at 100 Hz elicited at −70 mV, close to the neuronal resting membrane potential, to mimic physiological conditions. Following HFS, the amplitude of eIPSCs was immediately reduced, as was outward I_tonic_, but these values reached their peak after 15 min (Fig. 2*A,C,D*). During control conditions, the average eIPSC peak amplitude was 41±4.3 pA, corresponding to a peak conductance of 732.1±25.4 pS (n = 24) and I_tonic_ was 18.7±3.3 pA (n = 22). The average eIPSC rise time was 1.38±0.17 ms and the decay was fitted by a double exponential function with a Tau_1_ of 7.1±1 ms and Tau_2_ of 43.8±7 ms. Fifteen minutes after HFS, the eIPSC amplitude was 15.9±1.9 pA (P<0.001; Fig. 2*C,D*, n = 12), the peak conductance of eIPSCs was 322.1±18.7 pS (P<1.8×10^−5^) and outward I_tonic_ was decreased to 6.0±1.2 pA (P<0.00083, n = 10; Fig. 2*E,F*). The kinetics of the IPSCs were not significantly changed with a rise time of 1.6±0.21 ms (P = 0.32) and a decay Tau_1_ of 7±2.3 ms and a Tau_2_ of 43±4.5 ms. In five cells where stable recordings were maintained for over one hour, eIPSC amplitude partially recovered to 82.8±5.1% and the I_tonic_ to 80±3.1% of control in 40±6.5 minutes. Therefore, this form of transient inhibition of GABA_A_-receptor mediated responses is not a form of LTD.

To exclude a possible role of Group II and Group III mGlu receptors on this form of disinhibition we repeated experiments using a cocktail of antagonists that included LY341495 (100 µM), a broad spectrum inhibitor of mGlu receptors at this concentration. After a 10 min control period we delivered a HFS (Fig. 3). Following HFS, the amplitude of eIPSCs was immediately reduced, as was the outward I_tonic_, but these values reached their peak after 15 min (Fig. 3*A,C,D*). During control conditions, the average eIPSC peak amplitude was 45.2±4.2 pA, and I_tonic_ was 19.7±4.5 pA (n = 4). Fifteen minutes after HFS, the eIPSC amplitude was 20.1±3.6 pA (P<0.001; Fig. 3*C,D*, n = 4) and outward I_tonic_ was decreased to 7.6±3.1 pA (P<0.0001, n = 4; Fig. 3*E,F*).The differences in the percentage decrease for both phasic and tonic current in the presence of additional LY341495 are not statistically significant in comparison with the experiments without LY341495 (P>0.3).

To confirm that the inhibition is not the result of intracellular dialysis resulting from the whole-cell recording configuration, we performed experiments using the perforated patch-clamp technique [21]. With this approach, HFS still induced a decrease in eIPSCs similar to that obtained using the conventional whole-cell recording technique, with a peak current decrease of 52.1±1.4%, P<0.05 and a reduction in I_tonic_ by 29.7±1.1%, P<0.01 (n = 5, data not shown).

### Inhibition of Phasic and Tonic GABA_A_ Responses in Granule Cells is Mediated by GABA_B_ Receptors

Inhibitory neurotransmission has been reported to undergo negative modulation through GABA_B_ receptors in various brain areas [27]. When slices were pre-incubated for 30 minutes and perfused with the GABA_B_ receptor antagonist CGP35348 (50 µM), HFS (10 pulses at 100 Hz) no longer led to a significant change in eIPSCs and I_tonic_ with whole cell recording (Fig. 4*A*). In the presence of CGP35348, eIPSCs and I_tonic_ were no longer reduced (from 32.9±7.3 to 29.9±6.1 pA, P>0.3, n = 12, Fig. 4*A–D*) and (14.4±0.2 to 12.2±1.2 pA, P>0.8, n = 12, Fig. 4*E,F*), respectively. Thus, GABA_B_ receptors mediate the reduction in GABA_A_ responses in granule cells. These data also further confirm that run-down of eIPSCs is not confounding our data.

Application of CGP35348 (50 µM) alone had no significant effect on eIPSC amplitude (from 27.3±9.3 to 34.2±7.3 pA, P>0.7, n = 7) and I_tonic_ (from 21.7±1.3 to 23.0±4.2 pA, P>0.5, n = 7; data not shown), indicating that during low frequency stimulation ambient GABA does not affect the modulation of phasic and tonic responses mediated by postsynaptic GABA_B_ receptors in granule cells [28].

### Pharmacological Activation of GABA_B_ Receptors Mimics the Effect of HFS

We next examined whether we could reproduce the GABA_B_ receptor-dependent effects induced by HFS with pharmacological activation of GABA_B_ receptors. Application of the GABA_B_ receptor agonist baclofen (30 µM) for 30 minutes significantly decreased the amplitude of eIPSCs evoked at 0.1 Hz (from 28.3±3.8 to 11.6±1.7 pA, P<0.001, n = 8, Fig. 5*A–D*) and the I_tonic_ (from 20.2±8.2 to 11.4±6.8 pA, P<0.01, n = 8, Fig. 5*E,F*). Preincubation followed by bath-application of CGP35348, a selective GABA_B_-receptor antagonist, prevented the reduction by baclofen of eIPSC amplitude (from 38.3±6.6 to 39.2±2.7 pA, P>0.6, n = 5) and for I_tonic_ (from 25.5±2.9 to 21.3±4.4 pA, P>0.1, n = 5, Fig. 5*B,D,F*).

### G Protein-dependent Mechanism of GABA_A_ Receptor Modulation in Granule Cells

CGP35348 is an antagonist with preferential selectivity for postsynaptic GABA_B_ receptors [29], [30], [31]. We therefore performed experiments to confirm that the GABA_B_ receptors responsible for the inhibition of GABA_A_ receptor-mediated responses are localized postsynaptically. GABA_B_ receptor function was blocked specifically in the postsynaptic granule cell by disrupting G-protein-dependent signaling with GDPβS (1 mM), a non-hydrolyzable analog of GDP [32], introduced into cells by passive diffusion from the intracellular patch pipette. Because of the relatively fast dialysis of granule cells with patch pipettes [12], [33] it was possible to assess the effect of GDPβS in the first minutes after establishing the whole cell configuration. Furthermore, when a HFS was applied after 10 minutes of dialysis, the inhibition of eIPSCs was no longer observed. The I_tonic_ rapidly decreased in the first minute after establishing the whole cell configuration (from 22.9±4.8 to 7.9±2.6 pA, P<0.01, n = 5): therefore it was not possible to evaluate the effect of HFS (Fig. 6*A*). The tonic current decrease during intracellular dialysis by GDPβS before HFS is likely to reflect the blockade of G protein-sensitive ion channels that contribute to the generation of the resting potential, as described previously [34]. Introduction of GDPβS into granule cells also reduced the effects of GABA_B_ receptor activation in response to baclofen application (from 43.8±7.2 to 36.8±3.7 pA, P>0.2, n = 4, Fig. 6*E,F*). Therefore, the transduction mechanism linking GABA_B_ receptor activation to GABA_A_ phasic current involves G-protein dependent activation in the postsynaptic membrane.

### Activation of Postsynaptic GABA_B_ Receptors is Sufficient to Inhibit GABA_A_ Receptor-mediated Responses in Granule Cells

To verify that selective activation of postsynaptic GABA_B_ receptors is sufficient to inhibit granule cell eIPSCs, we blocked synaptic transmission by superfusing slices with TTX (1 µM) and activated postsynaptic GABA_A_ receptors on a granule cells by puff application of muscimol (pipette concentration: 100 µM). Again, application of baclofen (30 µM) for 5 min significantly reduced the amplitude of the chloride currents induced in granule cells (from 30.5±6.3 to 15.1±4.7 pA, P<0.008, n = 4, Fig. 7). This effect recovered after washout of baclofen (89.2±7.9%, n = 4).

## Discussion

Our main finding is that high frequency activation of Golgi cells leads to transient inhibition of phasic and tonic GABA_A_ receptor currents recorded in granule cells. This effect is mediated by GABA_B_ receptors, as it is blocked by the selective antagonist CGP35348, and is mimicked by bath-application of baclofen. CGP35348 had no effect on its own, indicating that ambient GABA concentration in cerebellar slices is insufficient to contribute to the depression of inhibition. Our data indicate that the inhibition of GABA_A_ responses is mediated primarily by postsynaptic GABA_B_ receptors, because 1) inactivation of G-proteins in the postsynaptic granule cell significantly reduced the effect, and 2) GABA_A_ receptor-mediated responses selectively induced in granule cells were depressed following the activation of GABA_B_ receptors.

### GABA_B_ Receptor-mediated Long-term Modulation of Inhibitory Responses

Although synaptic plasticity was originally characterized at excitatory synapses, it has long been known that GABAergic synapses can also undergo activity-dependent modification [35], [36]. Subsequent investigations identified LTP and LTD at inhibitory synapses throughout the brain, in some cases involving a presynaptic, in others, a postsynaptic mechanism of action [27]. In most of these studies it was shown that activation of pre or postsynaptic NMDA receptors initiates a complex signaling cascade culminating in the modulation of GABA_A_ receptor function. However, at inhibitory synapses in visual cortex [37], auditory brainstem [38], [39], and hippocampus [40], GABA_B_ receptors were identified as the triggers of plasticity. In general, GABA_B_ receptors are expressed both at presynaptic terminals as well as in the somatodendritic compartment of neurons, where they regulate myriad functions through numerous G-protein coupled signal transduction pathways [41]. Interestingly, in a previous study we observed a further example of modulation by postsynaptic GABA_B_ receptors of cerebellar properties in which GABA_B_ receptor action reduced a constitutive inwardly rectifying conductance in cerebellar granule cells [12].

In the cerebellar glomerulus, GABA_B_ receptors are expressed in both presynaptic Golgi cells [11] as well as in postsynaptic granule cells [42], [43]. We have previously shown that presynaptic GABA_B_ receptors on Golgi cell terminals are tonically activated by ambient GABA resulting in a negative modulation of neurotransmitter release [13]. In contrast, in the present study we found no evidence for tonic activation of the postsynaptic GABA_B_ receptors in granule cells. At present, the only evidence we have with regard to the transduction pathway is that the first step requires activation of a G-protein (Fig. 5). However, in a previous paper we showed that the modulation by GABA_B_ receptors of inwardly rectifying potassium channels depends on protein phosphatase 2A [12]. Furthermore, several studies in other brain regions have shown that phosphatases [44] and kinases [45], [46] regulate the function of postsynaptic GABA_A_ receptors.

### A GABAergic Paradox at the Golgi-cell-granule Cell Synapse: from Inhibition to Excitation

Some unusual features are emerging from *in vivo* studies on cerebellar circuits during sensory information processing. High-frequency “bursty” firing, a common feature in granule cells [47] leads to a decrease in Golgi cell firing through activation of mGlu2 receptors [4]. This contradicts the classical view that granule cells serve only to excite Golgi cells. The activation of metabotropic glutamatergic and metabotropic GABA_B_ receptors with high frequency discharge thus leads to paradoxical effects at the granule cell to Golgi cell synapse [4] and at the Golgi cell to-granule cell synapse (our data), indicating more complex interactions in the cerebellar glomerulus than previously assumed.

Golgi cells reside in the granular layer and are spontaneously active thus providing the sole source of inhibition for granule cells. At low frequency background firing rates (∼4 Hz), Golgi cells function like typical inhibitory interneurons to induce both phasic and tonic outward currents, whereas during high frequency firing, as occurs during sensory stimulation, spill-over in the cerebellar glomerulus and activation of GABA_B_ receptors can inhibit phasic and tonic responses mediated by GABA_A_ receptors. This GABA_B_ receptor-mediated inhibition of GABAergic transmission that we have characterized is likely to be of physiological significance in regulating cerebellar circuit function. We observed the effect with a train of 10 impulses at 100 Hz, which is in keeping with *in vivo* responses measured in Golgi cells [3], [16]. Synaptic inhibition at the mossy fiber input stage to the cerebellum is generated by short high frequency Golgi cell bursts [14], [15], [16], which typically consist of fast and precise responses that then slowly decrease over about 100 ms [48], [49].

The switch from an inhibitory to an excitatory action occurs when a Golgi cell discharges with a high frequency burst. With low frequency discharge, a Golgi cells inhibits granule cells through both phasic and tonic inhibition leading to strong lateral inhibition. In contrast, high frequency Golgi cell discharge has two distinct effects: 1) the system acts as a high-pass filter, allowing granule cell input to be processed by Purkinje cells. This effect is described in [50], figure 5*B and C*. High-frequency bursts transmitted through the granular layer are not reduced by inhibition. Thus, Golgi cell activity does not prevail over mossy fiber excitation during bursting discharge. This effect is in line with *in vivo* observations of protracted granule cell firing in response to sustained sensory stimuli [51], [52], [53]. These findings indicate that Golgi cells act as an activity-dependent gate, which permits cerebellar input to Purkinje cells to bypass Golgi cell inhibition when input is strong, and 2) the system acts as spatial filter. Spatial organization can play a critical role for distributed signal processing in neuronal networks. Lateral inhibition exerted by Golgi cells at low frequency results in a patchy configuration of granule cell activity [54] with limited dispersion of activity because of inhibition through the Golgi cell loop. Consequently, granular layer responses are spatially organized so that inhibition is strongest in areas of the granular layer surrounding (or adiacent to) granule cells activated by mossy fiber stimulation. With high frequency stimulation, Golgi cell inhibition as well as lateral inhibition is decreased, suggesting a change in the spatial properties of incoming information.

Previous studies on GABA_B_ receptor function in the cerebellar glomerulus have shown that their activation enhances excitatory transmission from mossy fibers to granule cells by inhibiting an inward rectifier in granule cells [12] and by decreasing release probability at GABAergic Golgi cell terminals [13]. Our present finding of GABA_B_ receptor-mediated disinhibition constitutes a third mechanism to promote glomerular excitatory transmission in the cerebellum.

## References

[1] Brickley SG, Cull-Candy SG, Farrant M (1999) Single-channel properties of synaptic and extrasynaptic GABAA receptors suggest differential targeting of receptor subtypes. J Neurosci 19: 2960–2973. 10191314 10.1523/JNEUROSCI.19-08-02960.1999 PMC6782265

[2] Farrant M, Nusser Z (2005) Variations on an inhibitory theme: phasic and tonic activation of GABAA receptors. Nat Rev Neurosci 6: 215–229. 15738957 10.1038/nrn1625

[3] Heine SA, Highstein SM, Blazquez PM (2010) Golgi cells operate as state-specific temporal filters at the input stage of the cerebellar cortex. J Neurosci 30: 17004–17014. 21159970 10.1523/JNEUROSCI.3513-10.2010 PMC3073632

[4] Holtzman T, Sivam V, Zhao T, Frey O, van der Wal PD, et al (2011) Multiple extra-synaptic spillover mechanisms regulate prolonged activity in cerebellar Golgi cell-granule cell loops. J Physiol 589: 3837–3854. 21669981 10.1113/jphysiol.2011.207167 PMC3171889

[5] Brickley SG, Cull-Candy SG, Farrant M (1996) Development of a tonic form of synaptic inhibition in rat cerebellar granule cells resulting from persistent activation of GABA receptors. J Physiol 497: 753–759. 9003560 10.1113/jphysiol.1996.sp021806 PMC1160971

[6] Wall MJ, Usowicz MM (1997) Development of action potential-dependent and independent spontaneous GABAA receptor-mediated currents in granule cells of postnatal rat cerebellum. Eur J Neurosci 3: 533–548. 10.1111/j.1460-9568.1997.tb01630.x 9104595

[7] Capogna M, Pearce RA (2011) GABA A,slow: causes and consequences. Trends Neurosci 34: 101–112. 21145601 10.1016/j.tins.2010.10.005

[8] Rossi DJ, Hamann M (1998) Spillover-mediated transmission at inhibitory synapses promoted by high affinity alpha6 subunit GABA_A_ receptors and glomerular geometry. Neuron 20: 783–795. 9581769 10.1016/s0896-6273(00)81016-8

[9] Hamann M, Rossi DJ, Attwell D (2002) Tonic and spillover inhibition of granule cells control information flow through cerebellar cortex. Neuron 33: 625–633. 11856535 10.1016/s0896-6273(02)00593-7

[10] Rossi DJ, Hamann M, Attwell D (2003) Multiple modes of GABAergic inhibition of rat cerebellar granule cells. J Physiol 548: 97–110. 12588900 10.1113/jphysiol.2002.036459 PMC2342786

[11] Kulik A, Nakadate K, Nyiri G, Notomi K, Malitschek B, et al (2002) Distinct localization of GABAB receptors relative to synaptic sites in the rat cerebellum and ventrobasal thlamus. Eur J Neurosci 15: 291–307. 11849296 10.1046/j.0953-816x.2001.01855.x

[12] Rossi P, Mapelli L, Roggeri L, Gall D, de Kerchove d’Exaerde A, et al (2006) Inhibition of constitutive inward rectifier currents in cerebellar granule cells by pharmacological and synaptic activation of GABAB receptors. Eur J Neurosci 24: 419–432. 16903850 10.1111/j.1460-9568.2006.04914.x

[13] Mapelli L, Rossi P, Nieus T, D’Angelo E (2009) Tonic activation of GABAB receptors reduces release probability at inhibitory connections in the cerebellar glomerulus. J Neurophysiol 101: 3089–3099. 19339456 10.1152/jn.91190.2008

[14] Edgley SA, Lidierth M (1987) The discharges of cerebellar Golgi cells during locomotion in the cat. J Physiol 392: 315–332. 3446782 10.1113/jphysiol.1987.sp016782 PMC1192306

[15] Van Kan PL, Gibson AR, Houk JC (1993) Movement-related inputs to intermediate cerebellum of the monkey. J Neurophysiol 69: 74–94. 8433135 10.1152/jn.1993.69.1.74

[16] Vos BP, Volny-Luraghi A, De Schutter E (1999) Cerebellar Golgi cells in the rat: receptive fields and timing of responses to facial stimulation. Eur J Neurosci 11: 2621–2634. 10457161 10.1046/j.1460-9568.1999.00678.x

[17] D’Angelo E, Rossi P, Taglietti V (1993) Different proportions of N-methyl-D-aspartate receptor currents at the mossy fibre-granule cell synapse of developing rat cerebellum. Neuroscience 53: 121–130. 8097019 10.1016/0306-4522(93)90290-v

[18] D’Angelo E, De Filippi G, Rossi P, Taglietti V (1995) Synaptic excitation of individual rat cerebellar granule cells in situ: evidence for the role of NMDA receptors. J Physiol 484: 397–413. 7602534 10.1113/jphysiol.1995.sp020673 PMC1157902

[19] D’Angelo E, Rossi P, Armano S, Taglietti V (1999) Evidence for NMDA and mGlu receptor-dependent long-term potentiation of mossy-fiber-granule cell transmission in rat cerebellum. J Neurophysiol 81: 277–287. 9914288 10.1152/jn.1999.81.1.277

[20] Blanton MG, Lo Turco JJ, Kriegstein AR (1989) Whole cell recording from neurons in slices of reptilian and mammalian cerebral cortex. J Neurosci Methods 30: 203–210. 2607782 10.1016/0165-0270(89)90131-3

[21] Horn R, Marty A (1988) Muscarinic activation of ionic currents measured by a new whole-cell recording method. J Gen Physiol 92: 145–59. 2459299 10.1085/jgp.92.2.145 PMC2228899

[22] Silver RA, Cull-Candy SG, Takahashi T (1996) Non-NMDA glutamate receptor occupancy and open probability at a rat cerebellar synapse with single and multiple release sites. J Physiol 494: 231–250. 8814618 10.1113/jphysiol.1996.sp021487 PMC1160626

[23] Cathala L, Brickley S, Cull-Candy S, Farrant M (2003) Maturation of EPSCs and intrinsic membrane properties enhances precision at a cerebellar synapse. J Neurosci 23: 6074–6085. 12853426 10.1523/JNEUROSCI.23-14-06074.2003 PMC6740347

[24] Kaneda M, Farrant M, Cull-Candy SG (1995) Whole-cell and single-channel currents activated by GABA and glycine in granule cells of the rat cerebellum. J Physiol 485: 419–35. 7545231 10.1113/jphysiol.1995.sp020739 PMC1158002

[25] Tia S, Wang JF, Kotchabhakdi N, Vicini S (1996) Distinct deactivation and desensitization kinetics of recombinant GABAA receptors. Neuropharmacology 35: 1375–82. 9014154 10.1016/s0028-3908(96)00018-4

[26] Carta M, Mameli M, Valenzuela CF (2004) Alcohol enhances GABAergic transmission to cerebellar granule cells via an increase in Golgi cell excitability. J Neurosci 24: 3746–51. 15084654 10.1523/JNEUROSCI.0067-04.2004 PMC6729340

[27] Castillo PE, Chiu CQ, Carroll RC (2011) Long-term plasticity at inhibitory synapses. Curr Opin Neurobiol 2: 328–338. 10.1016/j.conb.2011.01.006 PMC3092861 21334194

[28] Lei S, McBain CJ (2003) GABAB receptor modulation of excitatory and inhibitory synaptic transmission onto rat CA3 hippocampal interneurons. J Physiol 546: 439–453. 12527730 10.1113/jphysiol.2002.034017 PMC2342507

[29] Deisz RA, Billard JM, Zieglgänsberger W (1997) Presynaptic and postsynaptic GABAB receptors of neocortical neurons of the rat in vitro: differences in pharmacology and ionic mechanisms. Synapse 25: 62–72. 8987149 10.1002/(SICI)1098-2396(199701)25:1<62::AID-SYN8>3.0.CO;2-D

[30] Morishita W, Sastry BR (1995) Pharmacological characterization of pre- and postsynaptic GABAB receptors in the deep nuclei of rat cerebellar slices. Neuroscience 68: 1127–1137. 8544987 10.1016/0306-4522(95)00206-x

[31] Yamada K, Yu B, Gallagher JP (1999) Different subtypes of GABAB receptors are present at pre- and postsynaptic sites within the rat dorsolateral septal nucleus. J Neurophysiol 81: 2875–83. 10368404 10.1152/jn.1999.81.6.2875

[32] Eckstein F, Cassel D, Levkovitz H, Lowe M, Selinger Z (1979) Guanosine 5′-O-(2-thiodiphosphate). An inhibitor of adenylate cyclase stimulation by guanine nucleotides and fluoride ions. J Biol Chem 19: 9829–9834. 489574

[33] Gall D, Prestori F, Sola E, D’Errico A, Roussel C, et al (2005) Intracellular calcium regulation by burst discharge determines bidirectional long-term synaptic plasticity at the cerebellum input stage. J Neurosci 25: 4813–4822. 15888657 10.1523/JNEUROSCI.0410-05.2005 PMC6724778

[34] Lüscher C, Slesinger PA (2010) Emerging roles for G protein-gated inwardly rectifying potassium (GIRK) channels in health and disease. Nat Rev Neurosci. 11: 301–15. 10.1038/nrn2834 PMC3052907 20389305

[35] Stelzer A, Slater NT, ten Bruggencate G (1987) Activation of NMDA receptors blocks GABAergic inhibition in an in vitro model of epilepsy. Nature 326: 698–701. 2882427 10.1038/326698a0

[36] Komatsu Y, Iwakiri M (1993) Long-term modification of inhibitory synaptic transmission in developing visual cortex. Neuroreport 7: 907–910. 10.1097/00001756-199307000-00017 8103683

[37] Komatsu Y (1996) GABAB receptors, monoamine receptors, and postsynaptic inositol trisphosphate-induced Ca2+ release are involved in the induction of long-term potentiation at visual cortical inhibitory synapses. J Neurosci 20: 6342–6352. 10.1523/JNEUROSCI.16-20-06342.1996 PMC6578924 8815913

[38] Chang EH, Kotak VC, Sanes DH (2003) Long-term depression of synaptic inhibition is expressed postsynaptically in the developing auditory system. J Neurophysiol 90: 1479–1488. 12761279 10.1152/jn.00386.2003

[39] Kotak VC, DiMattina C, Sanes DH (2001) GABAB and Trk signaling mediates long-lasting inhibitory synaptic depression. J Neurophysiol 86: 536–540. 11431532 10.1152/jn.2001.86.1.536

[40] Patenaude C, Chapman CA, Bertrand S, Congar P, Lacaille JC (2003) GABAB receptor-and metabotropic glutamate receptor-dependent cooperative long-term potentiation of rat hippocampal GABAA synaptic transmission. J Physiol 553: 155–167. 12963794 10.1113/jphysiol.2003.049015 PMC2343476

[41] Misgeld U, Bijak M, Jarolimek W (1995) A physiological role for GABAB receptors and the effects of baclofen in the mammalian central nervous system. Prog Neurobiol 46: 423–462. 8532848 10.1016/0301-0082(95)00012-k

[42] Ige AO, Bolam JP, Billinton A, White JH, Marshall FH, et al (2000) Cellular and sub-cellular localisation of GABA(B1) and GABA(B2) receptor proteins in the rat cerebellum. Brain Res Mol Brain Res 83: 72–80. 11072097 10.1016/s0169-328x(00)00199-6

[43] Liang F, Hatanaka Y, Saito H, Yamamori T, Hashikawa T (2000) Differential expression of gamma-aminobutyric acid type B receptor-1a and -1b mRNA variants in GABA and non-GABAergic neurons of the rat brain. J Comp Neurol 416: 475–495. 10660879

[44] Chen QX, Wong RK (1995) Suppression of GABAA receptor responses by NMDA application in hippocampal neurones acutely isolated from the adult guinea-pig. J Physiol 482: 353–62. 7714826 10.1113/jphysiol.1995.sp020522 PMC1157733

[45] Abramian AM, Comenencia-Ortiz E, Vithlani M, Tretter EV, Sieghart W, et al (2010) Protein kinase C phosphorylation regulates membrane insertion of GABAA receptor subtypes that mediate tonic inhibition. J Biol Chem. 285: 41795–805. 10.1074/jbc.M110.149229 PMC3009907 20940303

[46] Kia A, Ribeiro F, Nelson R, Gavrilovici C, Ferguson SS, et al (2011) Kindling alters neurosteroid-induced modulation of phasic and tonic GABAA receptor-mediated currents: role of phosphorylation. J Neurochem. 116 1043–56. 10.1111/j.1471-4159.2010.07156.x 21175618

[47] Bengtsson F, Jörntell H (2009) Sensory transmission in cerebellar granule cells relies on similarly coded mossy fiber inputs. Proc Natl Acad Sci U S A 106: 2389–2394. 19164536 10.1073/pnas.0808428106 PMC2650166

[48] Eccles JC, Ito M, Szentagothai J (1967) The Cerebellum as a Neuronal Machine. Berlin: Springer.

[49] Maffei A, Prestori F, Rossi P, Taglietti V, D’Angelo E (2002) Presynaptic current changes at the mossy fiber-granule cell synapse of cerebellum during LTP. J Neurophysiol 88: 627–638. 12163516 10.1152/jn.2002.88.2.627

[50] Mapelli J, Gandolfi D, D’Angelo E (2010) Combinatorial responses controlled by synaptic inhibition in the cerebellum granular layer. J Neurophysiol 103: 250–61. 19906881 10.1152/jn.00642.2009

[51] Chadderton P, Margrie TW, Häusser M (2004) Integration of quanta in cerebellar granule cells during sensory processing. Nature 428: 856–60. 15103377 10.1038/nature02442

[52] Jörntell H, Ekerot CF (2002) Reciprocal bidirectional plasticity of parallel fiber receptive fields in cerebellar Purkinje cells and their afferent interneurons. Neuron 34: 797–80. 12062025 10.1016/s0896-6273(02)00713-4

[53] Rancz EA, Ishikawa T, Duguid I, Chadderton P, Mahon S, et al (2007) High-fidelity transmission of sensory information by single cerebellar mossy fibre boutons. Nature 450: 1245–8. 18097412 10.1038/nature05995 PMC5881887

[54] Mapelli J, D’Angelo E (2007) The spatial organization of long-term synaptic plasticity at the input stage of cerebellum. J Neurosci 27: 1285–96. 17287503 10.1523/JNEUROSCI.4873-06.2007 PMC6673576

