# Correction: Golgi Cell-Mediated Activation of Postsynaptic GABA_B_ Receptors Induces Disinhibition of the Golgi Cell-Granule Cell Synapse in Rat Cerebellum

**DOI:** 10.1371/annotation/2a19c750-eeb2-491a-9e6d-6be98c380984

**Published:** 2013-01-29

**Authors:** Federico Brandalise, Lisa Mapelli, Urs Gerber, Paola Rossi

Following an investigation at the University of Pavia, it has been established that Lisa Mapelli made a substantial contribution to the work reported in this study and that her contribution fulfills the requirements for authorship for the article. This Correction is issued to reflect her contribution and include Dr Mapelli as second author of the article, the author list should therefore be revised as follows:

Federico Brandalise, Lisa Mapelli, Urs Gerber, Paola Rossi

Dr Mapelli contributed to the collection and analysis of data and the processing of the figures and has no competing interests. 

